# A systematic review of the health, social and financial impacts of welfare rights advice delivered in healthcare settings

**DOI:** 10.1186/1471-2458-6-81

**Published:** 2006-03-29

**Authors:** Jean Adams, Martin White, Suzanne Moffatt, Denise Howel, Joan Mackintosh

**Affiliations:** 1 Public Health Research Group, School of Population and Health Sciences, University of Newcastle upon Tyne, Newcastle upon Tyne, UK

## Abstract

**Background:**

Socio-economic variations in health, including variations in health according to wealth and income, have been widely reported. A potential method of improving the health of the most deprived groups is to increase their income. State funded welfare programmes of financial benefits and benefits in kind are common in developed countries. However, there is evidence of widespread under claiming of welfare benefits by those eligible for them. One method of exploring the health effects of income supplementation is, therefore, to measure the health effects of welfare benefit maximisation programmes. We conducted a systematic review of the health, social and financial impacts of welfare rights advice delivered in healthcare settings.

**Methods:**

Published and unpublished literature was accessed through searches of electronic databases, websites and an internet search engine; hand searches of journals; suggestions from experts; and reference lists of relevant publications. Data on the intervention delivered, evaluation performed, and outcome data on health, social and economic measures were abstracted and assessed by pairs of independent reviewers. Results are reported in narrative form.

**Results:**

55 studies were included in the review. Only seven studies included a comparison or control group. There was evidence that welfare rights advice delivered in healthcare settings results in financial benefits. There was little evidence that the advice resulted in measurable health or social benefits. This is primarily due to lack of good quality evidence, rather than evidence of an absence of effect.

**Conclusion:**

There are good theoretical reasons why income supplementation should improve health, but currently little evidence of adequate robustness and quality to indicate that the impact goes beyond increasing income.

## Background

Socio-economic variations in health, including variations in health according to wealth and income, have been widely reported [1-4]. However, interventions to overcome socio-economic variations in health have achieved little success[5,6]. One potential method of improving the health of the most deprived groups is to increase their income. Despite a number of income supplementation experiments – particularly in the USA in the 1960s and 1970s – little investigation of the impact of these experiments on health has been performed[7].

State funded welfare programmes of financial benefits and benefits in kind for, amongst others, the unemployed, the elderly and the sick are common in developed countries. However, there is evidence of widespread under claiming of welfare benefits by those eligible for them, with take up of income related benefits in the UK around 80% in 2002[8]. Take up rates in the rest of Europe are around 40–80% with generally lower rates in the USA[9]. One method of exploring the health effects of income supplementation is, therefore, to measure the health effects of welfare benefit maximisation programmes[7].

Efforts to provide advice on claiming welfare benefits are increasingly being made in the UK[10]. In general, 'welfare rights advice' involves review of eligibility for welfare benefits and active assistance with claims for any benefits to which the client is found to be entitled. Active assistance includes help with completing forms, telephone calls, obtaining letters of support and references, and attendance in person at benefit tribunals. Welfare rights advisors are also often able to offer debt counselling and legal advice, or refer to other appropriate agencies. In the UK, where the majority of welfare rights advice programmes are based, advice is primarily offered through local government, Citizens Advice Bureaux (CAB – a voluntary organisation that "helps people resolve their legal, money and other problems by providing free information and advice"[11] from community locations) or primary care, with clients accessing the services either through self referral, referral from another agency, or a combination of both.

Welfare rights advice services delivered at, or through, primary care premises work within a holistic model of primary health care that "involves continuity of care, health promotion and education, integration of prevention with sick care, a concern for population as well as individual health, community involvement and the use of appropriate technology"[12]. In the UK, all individuals who have been legally resident for at least six months are entitled to be registered with a local primary care practice and receive free treatment there. As over 98% of the population is registered with a primary care practice[13], primary care provides a setting in which the great majority of the population can be accessed.

Given the increasing interest in this area, particularly in the UK, the funding that is now being committed to it by primary care organisations and local authorities, and the opportunity it offers to assess the impact of income supplementation on health, it is timely to bring together the available evidence on the impacts of welfare rights advice delivered in healthcare settings. Two previous reviews have focused on welfare rights advice in healthcare settings[14,15]. However, neither of these took a systematic approach to literature searching and were primarily descriptions of the different programmes on offer, rather than an assessment of the impacts of these.

We performed a systematic review in order to answer the question: what are the health, social and financial impacts of welfare rights advice delivered in healthcare settings?

## Methods

### Search strategy

**Figure 1:**
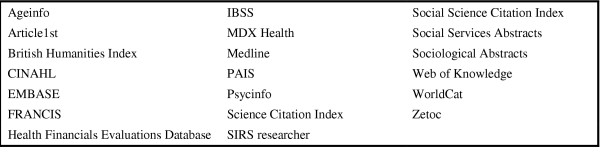
Box 1. Electronic databases searched.

**Figure 2:**
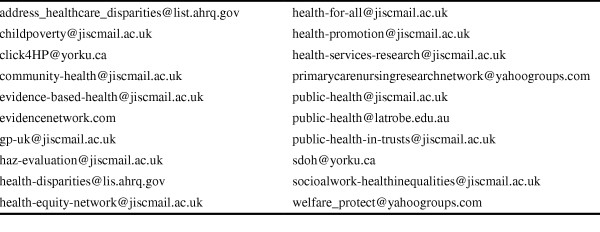
Box 2. Email distribution lists sent requests for information.

**Figure 3:**
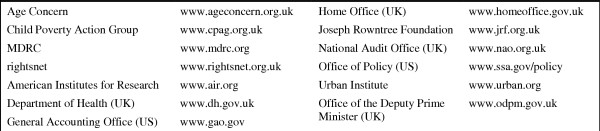
Box 3. Websites hand searched for relevant publications.

The following strategies were used (by JA) to find and access potentially relevant studies for consideration for inclusion in the review:

1. *Searches of electronic databases: *the keyword search "(welfare OR benefit OR social welfare OR citizen OR money OR assistance) AND (advice OR right OR prescrip$ OR counsel$)" was used to search the electronic databases listed in Box 1 (see Figure 1) (where $ = wildcard symbol). All available years of all databases were searched up to and including October 2004.

2. *Hand searches of specific journals*: the electronic contents pages of *Health and Social Care in the Community *(volumes 6–12, 1998–2004), and the *Journal of Social Policy *(volumes 26–33, 1997–2004) were scanned to identify relevant publications[16]. These journals were chosen because of their relevance to the subject area and the perception that substantial relevant work had been published in them.

3. *Searches of internet search engine: *searches were made of the internet search engine Google  using the same strategies as above. The first 100 results returned by each search strategy were scanned for relevance and those judged to be potentially relevant followed up.

4. *Suggestions from experts and those working in the field*: requests for help with accessing relevant literature were sent to relevant e-mail distribution lists (listed in Box 2 – see Figure 2), posted on the rightsnet.org.uk discussion forum and published in the 'trade magazines' *Poverty *and *Welfare Rights Bulletin*. 'Experts' – identified as such either by frequent publication in the area, or through personal contacts of the research team – were also contacted directly and asked for help with identifying relevant literature or providing further contacts[17].

5. *Searches of specific websites: *the websites of a number of specific organisations that sponsor and conduct social policy research (listed in Box 3 – see Figure 3) were searched to identify publications of interest.

6. *Reference lists from relevant studies: *the reference lists of all studies assessed to be relevant were scanned to identify other relevant work, as were the reference lists of previous reviews in this area[14,15].

7. *Science Citation Index and Social Science Citation Index: *citation searches of the Science Citation Index and Social Science Citation Index were performed to identify all citations of studies identified as relevant.

8. *Author searches: *searches for other articles by all authors of articles included in the review were performed in Medline and Health Management Information Consortium (the two databases that provided the greatest number of relevant hits) for all available years up to and including October 2004.

### Inclusion and exclusion criteria for studies included in the review

Studies were considered relevant and included in the review if they reported an evaluation of welfare rights advice in a healthcare setting in terms of health, social or financial outcomes. We defined 'welfare rights advice' as expert advice concerning entitlement to and claims for welfare benefits. 'Healthcare settings' were defined as health related buildings – including primary, secondary or tertiary care centres – or where clients were identified through primary, secondary or tertiary care patient lists.

A preliminary scoping review revealed that: there is substantial 'grey literature' in this area; the main study design used is uncontrolled before and after studies; and outcome variables studied vary widely. In order to provide an overview of the wide variety of impacts of welfare rights advice delivered in healthcare settings, we did not restrict our review to any particular outcomes, study design, methods, study population or place of publication (i.e. studies not published in peer reviewed journals were not necessarily excluded). Although searches were conducted in English, no *a priori *exclusions were made based on the language of publication. However, we did not identify any potentially relevant studies that were not written in English.

The process of determining whether studies should be included in the review was made by one reviewer (JA) in the majority of cases. The review team discussed any cases where doubt concerning inclusion remained after retrieval of reports.

### Data abstraction

Data were abstracted from reports and papers ("studies") in the review using a structured proforma. Data collected included: descriptive details of interventions delivered and evaluations performed, and outcome data on all financial, social and health outcomes measured. Data abstraction from each report was performed independently by pairs of reviewers with information entered onto a Microsoft Access database for recording and analysis. In cases where reviewers were found to disagree about the data abstracted, reviewers met to discuss disagreements. If agreement could not be reached, the whole review team was asked to consider the issue and reach a consensus.

Where investigators reported data on the same outcome at a number of different follow up times, information from all follow ups was abstracted and reported. Where information on a number of different outcomes was reported from the same project, information on all outcomes reported was abstracted and the results presented to highlight that these are not independent findings. When we retrieved both an internal report and peer reviewed paper on the same project, both documents were scrutinised and if discrepancies were found, results reported in peer-reviewed journals were used in our assessment.

### Assessment of study quality

As the majority of quantitative evaluations of welfare rights advice delivered in healthcare settings use a simple before and after design (6 of 8 studies that reported data on health and social outcomes employed a before and after design, all 29 studies that reported data on financial outcomes employed a before and after design), we felt it inappropriate to assess the quality of studies reported in terms of a formal scoring framework. Instead, we collected information on various aspects of methodology and report this in a descriptive analysis.

As with the quantitative evaluative work in this area, few qualitative studies, or components of studies, identified in the scoping review appeared to meet many of the quality standards for qualitative research that have been proposed[18,19]. As before, we did not apply any formal framework for determining quality in qualitative work. Instead, information on various aspects of methodology were recorded and are reported descriptively

### Analyses and reporting

Given the wide variety of studies that we anticipated including in the review, a formal meta-analysis was not planned and results are reported primarily in a narrative form according, as far as possible, to the schema proposed by Stroup et al (2000) – a checklist of topics that should be covered in meta-analyses of observational studies under the general headings of background, search strategy, methods, results, discussion and conclusions devised by an expert working group (The Meta-analysis Of Observational Studies in Epidemiology (MOOSE) Group)[20].

### Ethics and research governance

This review of published and publicly available literature did not require ethical approval.

## Results

### Search results

Results of electronic database searches for articles, citation searches and author searches are reported in Table 1, Table 2 and Table 3 respectively. Numerous reports were identified by responders to the requests for information. Overall, 55 different studies, considered to meet the inclusion criteria, were included in the review and are summarised in Table 4. Where single reports contained data on two or more projects that differed substantially in design[21,22], these different projects are reported as separate studies in the results. Table 5 lists those papers and reports retrieved but not included in the review with reasons for exclusion. Only one study included in the review was not UK based[23].

### Interventions delivered

Interventions delivered took a number of different forms. Some identification of who delivered the intervention was reported in 54 (98%) cases. In 30 (55%) instances all or some of the advice was delivered by employees of, or volunteers for, the CAB. In a further 22 (40%) studies all or some of the advice was delivered by welfare rights workers, officers and advisers – sometimes, but not always, explicitly identified as employees of local government.

The location where advice was delivered was reported in 54 (98%) cases. In 31 (57%) instances advice was delivered only in primary care premises such as general practice surgeries or health centres. In a further 16 (29%) cases advice was delivered in primary care premises along with one or more other locations, including clients' homes, hospitals and local CAB. Overall, 18 (33%) studies offered advice within clients' own homes – either exclusively or as an available option.

The referral system by which individuals gained access to the welfare rights advice was reported in 44 (80%) studies. In 32 (73%) studies referral could be from any member of the primary care team, a member of another relevant agency, via self referral from clients or via a combination of these modes. In 11 (25%) studies there were more formal eligibility criteria and invitational processes.

Criteria for who was eligible to receive the welfare rights advice given were reported in 31 (56%) studies. In 14 (45%) studies all patients registered at the general practice or practices participating in the project were eligible to receive advice. In a further 15 (48%) studies some sort of screening or sampling procedure was used to restrict eligibility to certain subgroups of the population – often those suffering from particular conditions or over a certain age. In two cases it was explicitly stated that welfare rights advice was only offered for a limited number of specified benefits (Attendance Allowance and Disability Living Allowance in both cases)[24,25].

The size of the population eligible to receive the advice given was reported in 17 (31%) studies. Eligible populations ranged in size from 1690 to 313 510 with a median of 23 039.

### Health and social outcomes – studies with a comparison or control group

Results from studies that reported the use of a comparison or control group are summarised in Table 6. Of the seven studies with a control or comparison group that reported non-financial outcomes, only one[23] randomly assigned individuals to the intervention or control group.

Outcome measures used included the Short Form 36 (SF-36 – a general health scale)[26,27], the Hospital Anxiety and Depression Scale (HADS – a questionnaire commonly used to screen for anxiety or depression)[28], the Measure Yourself Medical Outcome Profile scale (MYMOP – a patient generated wellbeing scale)[29], the Nottingham Health Profile (NHP – a quality of life scale)[30], and the Edinburgh Post-natal Depression Scale[31], as well as whether or not benefits had been applied for or received, and a variety of measures of use of health services. The size of intervention groups at follow up ranged from 13 to 303 with five studies reporting intervention group sizes at follow up of less than 70. Control or comparison group sizes at follow up ranged from 12 to 311 with five studies having control or comparison group sizes at follow up of less than 51. Follow up periods ranged from six to 12 months.

The majority of studies assessed the effect of the advice by comparing change in scores between baseline and follow up in the control or comparison group with the intervention group. Out of 72 separate comparisons reported, 11 (15%) were statistically significant at the 5% level including comparisons relating to SF36 vitality, SF36 mental health, SF36 bodily pain, SF36 role functioning emotional, SF36 mental health, NHP emotional reactions and the proportion of participants who had both applied for and received an award.

### Health and social outcomes – before-and-after study design

The six studies that reported non-financial results using recognised measurement scales and a before-and-after study design are summarised in Table 7. These studies used four different outcome measures – the SF36, HADS, MYMOP and NHP. Sample sizes included in follow up ranged from 22 to 244 with five out of six studies completing follow up on less than 55 individuals. Reported follow up periods ranged from six to 12 months. Out of 59 separate statistical comparisons reported, 6 (10%) were found to be significant – SF36 vitality, SF36 role functioning emotional, SF36 mental health, SF36 general health, NHP pain and NHP emotional reactions. Three studies, including one with a follow up sample size of 244 at six months and 200 at 12 months, reported no statistically significant comparisons at all.

Seven studies reported health and social results using in-house questionnaires with little evidence of validation. These are summarised in Table 8. These studies found consistently high levels of clients agreeing with statements concerning the positive impact of the advice on their health, quality of life and living situations.

### Health and social outcomes – qualitative studies

Aspects of the qualitative investigations within studies included in the review are summarised in Table 9. The 14 studies that reported qualitative data collected information from a variety of individuals including those who received advice, advice givers and primary care staff. Sample sizes ranged from six to 41. In 12 of the 14 (86%) studies, data were collected via interviews with participants whilst questionnaires were relied on in two (14%) cases. Six of 12 (50%) studies that reported a rationale for participant selection, gave a theoretical reason for participant selection, rather than reporting that selection was random, opportunistic or just those who responded to a postal questionnaire. The analytical approach used for drawing results from the data was reported in 10 (71%) cases.

**Figure 4:**
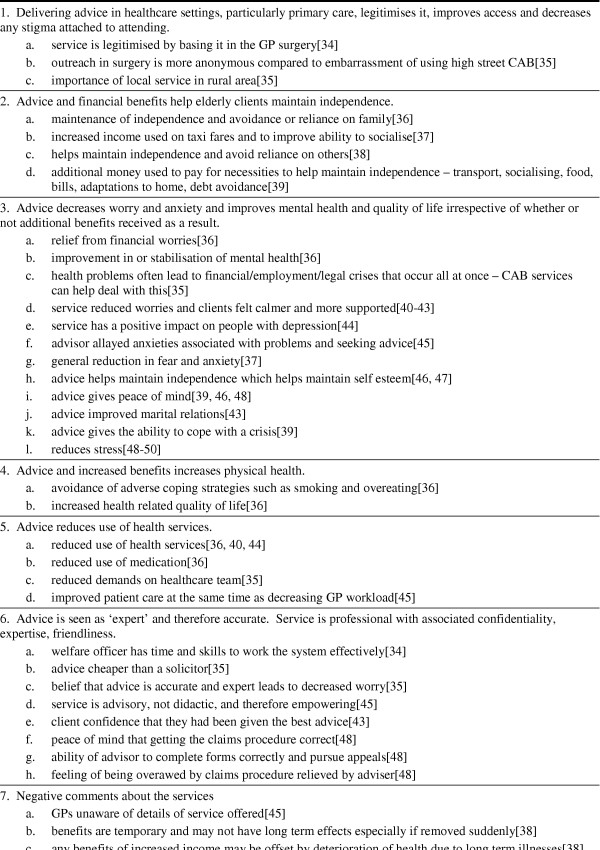
Box 4. Common areas identified in qualitative work.

Some of the common themes identified in the qualitative results are listed in Box 4 (see Figure 4). Money gained as a result of the advice was commonly reported as being spent on healthier food, avoidance of debt, household bills, transport and socialising. A number of negative issues concerning the advice were raised, primarily by general practitioners. These included the suggestion that the health benefits of increased welfare benefits may be temporary or offset by ongoing, irreversible, health deterioration.

### Financial outcomes

Data on either lump sums (generally back dated payments and arrears for the period between claim submission and claim approval) or recurring benefits or both gained as a result of the advice were reported in 28 cases (51%). Financial data from these studies are summarised in Table 10. Although a number of other studies reported some information on financial outcomes, this was often given as a combined figure of both lump sum payments and recurring benefits – making comparisons difficult. Furthermore, the specific benefits gained for clients was inconsistently reported and are not, therefore, reported here. The studies reporting analysable financial data gained a mean of £194 (US$353, €283) lump sum plus £832 (US$1514, €1215) per year in recurring benefits per client seen – a total of £1026 (US$1867, €1498) in the first year following the advice per client seen. As, the number of successful claimants was only reported in 17 (59%) cases where all other financial data were reported, we have not reported gains per successful claimant. As the number of successful claimants is likely to be less than the total number of clients seen, the actual financial benefit to those who successfully claimed is likely to be greater than the figures summarised here. Furthermore, a number of authors stated that their data did not include the outcomes of claims or appeals still pending at the time of reporting, making the definitive amount gained as a result of advice likely to be greater still.

## Discussion

### Summary of results

We found 55 studies reporting on the health, social and economic impact of welfare advice delivered in healthcare settings. The majority of these studies were grey literature, not published in peer reviewed journals, and were of limited scientific quality: full financial data were only reported in 50% of cases, less than 10% of studies used a control or comparison group to assess the impact of the advice, and qualitative approaches did not always reflect best practice. Only one study – based in the USA – included in the review was not UK based.

Amongst those studies included in the review, most welfare rights advice was delivered by CAB workers or local government welfare rights officers, most advice was delivered in primary care with around a third of studies offering advice in clients' homes. Few studies had restrictive eligibility criteria or referral procedures.

There was evidence that welfare rights advice delivered in healthcare settings leads to worthwhile financial benefits with a mean financial gain of £1026 per client seen in the year following advice amongst those studies reporting full financial data. This equates to around 9% of average individual gross income in the UK in 1999–2001[32]. However, this is by no means a precise estimate of typical gains: there was considerable variation in the gains reported and many studies identified that their data were incomplete with a number of claims still 'pending'.

Studies that included control or comparison groups tended to use non-specific measures of general health (e.g. SF36, NHP and HADS) and found few statistically significant differences between intervention and control or comparison groups. However, sample sizes were often small and follow up limited to a maximum of 12 months – likely to be too short a period to detect changes in health following changes in financial circumstances. Where statistically significant results were found, these tended to be in relation to measures of psychological or social, rather than physical, health. Qualitative methods were commonly used to assess both clients' and staff's perceptions of the impact of the advice. The advice was generally welcomed with extra money gained as a result of the advice commonly reported as being spent on household necessities and social activities.

### Limitations of review methods

The majority of the studies included in this review were grey literature not published in peer reviewed journals and were accessed via requests for information sent to email distribution lists. Although often of limited scientific quality, we included these studies in our review as they often included legitimate data on financial benefits of the intervention and let us describe the current scope of welfare rights advice as far as possible. Because grey literature is not comprehensively indexed, it is hard to be sure that we accessed all that is available, despite our use of a systematic approach to both literature searching and data abstraction[17]. In particular, we collected very little information from non-UK settings, despite sending requests for information to a number of international distribution lists. Whilst welfare rights advice may be rare outside the UK, it is also possible that it is described differently in different contexts and that the vocabulary used in our requests for information had little meaning for those outside the UK. We did not conduct searches of non-English language electronic databases or place posts in other languages to international email distribution lists. These additional techniques may have revealed additional relevant work from outside the UK.

The variations and limitations of methods used by the studies included in this review meant that it was inappropriate to perform formal meta-analysis. Similarly, limitations in data availability prevented us from performing potentially interesting comparisons of the cost of providing welfare rights advice versus the financial benefits gained for clients. The interpretation of our findings and conclusions that can be drawn are, therefore, more subjective than might be the case in other systematic reviews. In order to confirm that we were using the best possible methods, we considered performing our review under the umbrella of one of the evidence and review collaborations. However, there was no obvious appropriate review group within the Cochrane Collaboration for this sort of work. The Campbell Collaboration supports systematic reviews of behavioural, social and educational interventions but were unwilling to consider inclusion of any uncontrolled studies in our review. Although this would undoubtedly have increased the overall quality of studies included, we felt it would have led to a review that was not representative of the evidence base – which is largely of poor scientific quality, as described here. This problem has been previously described[12].

### Interpretation of results

Our review supports previous findings that the provision of welfare rights advice in healthcare settings is increasingly common in the UK[14,15] – although as these are non-statutory services, coverage is inevitable patchy. However, there was also some evidence that similar programmes can be provided in other settings with one study from the USA included in the review[23]. Whilst we have found substantial evidence that welfare rights advice in healthcare settings leads to financial benefits, there is little evidence that the advice leads to measurable health and social benefits. This is primarily due to absence of good quality evidence, rather than evidence of absence of an effect.

Whilst some sort of evaluation of welfare rights advice programmes is commonplace, the scientific rigour of these evaluations appears to be limited. Many of these advice services appear to operate in conditions of limited resources. Although performing some sort of evaluation of their service is frequently a requirement of funding, additional resources to support such evaluation and the skills to conduct it rigorously are scarce.

### Implications for policy, practice and research

There is now substantial evidence that welfare rights advice delivered in healthcare settings leads to financial benefits for clients – although typical levels cannot be precisely estimated. There is little need to conduct additional work to determine whether such advice has a financial effect, although further work is required to explore the characteristics of those most likely to benefit financially in order that such advice can be effectively targeted.

As there is little evidence either that welfare rights advice in healthcare settings does or does not have health and social effects, and this remains an intervention with theoretical potential to improve health, there is a need for further studies to examine these effects using robust methods. In particular, future work should: use randomised and controlled approaches; put careful consideration into the outcome measures to be used – general measures of health such as the SF36 may not be able to pick up subtle changes in psychological and social aspects of health; and make efforts to follow up participants over an appropriate time period – as the health and social effects of increased financial resources may take years, rather than months, to become apparent. There has been some discussion concerning the ethics of conducting randomised controlled trials of welfare rights advice interventions as it may be considered unethical to randomise some participants to a control group when there is good reason to believe that the intervention will lead to financial benefit for many participants[33]. However, if the control condition comprises 'usual care' and control group participants are free to seek out welfare rights advice from routine sources should they wish, it is not clear why such trials should necessarily be unethical.

There is also a need for evaluations of the effects of welfare rights advice in healthcare settings outside the UK. All welfare benefits systems are country specific and it can not be assumed that results for one country – such as the majority of those included in this review – are necessarily generalisable internationally. However, many of the conclusions of this review, in terms of how interventions are evaluated, will be applicable internationally.

## Conclusion

This review has revealed the poor quality of many evaluations of welfare rights advice in healthcare settings. If firm conclusions about the health and social effects of such advice are to be drawn, future evaluative work should be well resourced and carried out by those with appropriate skills. Those funding such programmes should think carefully about the benefits of requiring evaluations to be performed without providing additional resources and skills – poor quality evaluations could be argued to be a waste of money.

This review confirms that there is a substantial under claiming of welfare benefits amongst those referred to welfare rights advice services and that such services can go some way to resolving under claiming. However, there is currently little evidence of adequate robustness and quality to indicate that such services lead to health improvements.

## Competing interests

DH, JM, SM and MW have recently completed a pilot randomised controlled trial of welfare rights advice in primary care.

## Authors' contributions

MW and SM conceived the idea for this review. All authors contributed to protocol development. JA performed the literature searches, reviewed all studies found and drafted the manuscript. MW, SM, DH and JM provided second reviews for all studies included. All authors read and approve the final manuscript.

## Pre-publication history

The pre-publication history for this paper can be accessed here:



## Figures and Tables

**Table 1:** results of electronic database searches

**Database**	**Hits**	**Of some relevance**	**Included in review**
Ageinfo	5	1[34]	1[34]
British Humanities Index	67	0	0
CINAHL	99	6[35–40]	1[40]
Embase	141	7[25, 37, 41–45]	4[25, 42–44]
Health Management Information Consortium	38	14[14, 36–38, 40, 42, 43, 45–47]	4[14, 40, 42, 43]
Health Financials Evaluations Database	0	0	0
International Bibliography of the Social Sciences	113	0	0
MDX health	0	0	0
Medline	286	15[25, 34, 36, 38, 41–45, 48–53]	5[25, 34, 42–44]
PAISArchive	82	0	0
PAISInternational	83	2[54, 55]	0
PsycINFO	686	3[41, 53, 56]	0
Science citation index	150	8[25, 37, 41–45, 57]	5[25, 42–44, 57]
SIRS researcher	5	0	0
Social science citation index	237	7[36–38, 41–43, 45]	2[42, 43]
Social Services Abstracts	147	3[36, 38, 58]	0
Sociological Abstracts	293	2[59, 60]	0
Zetoc	0	0	0

**Table 2:** results of citation searches

**Article**	**Hits**	**Of some relevance**	**Included in review**
Abbott and Hobby (2000)[42]	3	3[36, 37, 61]	1[61]
Coppel et al (1999)[43]	7	7[36, 37, 42, 61–64]	3[42, 61, 63]
Cornwallis and O'Neil (1998)[65]	Journal (Hoolet) not listed		
Dow and Boaz (1994)[23]	4	1[66]	1[66]
Frost-Gaskin et al (2003)[66]	0	0	0
Galvin et al (2000)[67]	4	4[25, 36, 37, 61]	2[25, 61]
Greasley and Small (2005)	0	0	0
Hoskins and Smith (2002)[63]	2	1[68]	1[68]
Langley et al (2004)[25]	1	1[68]	1[68]
Memel and Gubbay (1999)[57]	2	2[24, 61]	2[24, 61]
Memel et al (2002)[24]	3	2[25, 68]	2[25, 68]
Middleton et al (1993)[69]	4	4[36, 37, 63, 64]	1[63]
Moffatt et al (2004)[70]	Journal (Critical Public Health) not listed		
Paris and Player (1993)[71]	21	14[36, 37, 43, 44, 61, 63, 64, 67, 68, 72–76]	7[43, 44, 61, 63, 67, 68, 72]
Powell et al (2004)[68]	0	0	0
Reading et al (2002)[72]	1	1[61]	1[61]
Sherratt et al (2000)[77]	Journal (Primary Healthcare Research and Development) not listed		
Toeg et al (2003)[61]	1	0	0
Veitch and Terry (1993)[44]	0	0	0

**Table 3:** results of author searches

	**Medline**			**Health Management Information Consortium**		
**Author**	**Hits**	**Of some relevance**	**Included in review**	**Hits**	**Of some relevance**	**Included in review**

Abbott, S	38	4[36, 37, 42, 78]	1[42]	3	1[42]	1[42]
Boaz, TL	9	1[23]	1[23]	0	0	0
Coppel, DH	1	1[43]	1[43]	3	0	0
Cornwallis, E	0	0	0	0	0	0
Dow, MG	17	1[23]	1[23]	0	0	0
Downey, D	45	0	0	1	0	0
Frost-Gaskin, M	1	1[66]	1[66]	0	0	0
Galvin, K	35	0	0	12	1[67]	1[67]
Greasley, P	8	0	0	6	0	0
Gubbay, D	3	2[25, 68]	2[25, 68]	0	0	0
Hehir, M	34	1[24]	1[24]	1	0	0
Henderson, C	147	1[66]	1[66]	17	0	0
Hewlett, S	21	3[24, 25, 68]	3[24, 25, 68]	3	0	0
Hobby, L	5	3		10	6[34, 36, 40, 42, 78, 79]	4[34, 40, 42, 79]
Hoskins, RA	12	1[63]	1[63]	5	2[63, 64]	1[63]
Hudson, E	42	0	0	2	0	0
Illife, S	85	1[61]	1[61]	2	0	0
Jackson, D	501	0	0	19	1[67]	1[67]
Jones, K	581	0	0	90	1[77]	1[77]
Kirwan, J	47	1[68]	1[68]	6	0	0
Langley, C	25	3[24, 25, 68]	3[24, 25, 68]	6	0	0
Lenihan, P	13	1[61]	1[61]	10	1[61]	1[61]
Means, R	13	1[68]	1[68]	63	0	0
Memel, D	6	1[68]	1[68]	5	0	0
Mercer, L	16	1[61]	1[61]	1	1[61]	1[61]
Middleton, P	51	0	0	7	1[77]	1[77]
Moffatt, S	29	0	0	2	0	0
O'Kelly, R	6	1[66]	1[66]	8	0	0
O'Neil, J	101	0	0	6	0	0
Packham, CK	11	1[43]	1[43]	1	0	0
Paris, JA	14	1[71]	1[71]	2	1[71]	1[71]
Player, D	11	1[71]	1[71]	13	1[71]	1[71]
Pollock, J	86	2[25, 68]	2[25, 68]	2	0	0
Powell, JE	57	1[68]	1[68]	22	0	0
Reading, R	28	1[80]	1[80]	14	0	0
Reynolds, S	106	1[72]	1[72]	13	0	0
Sharples, A	25	0	0	2	1[67]	1[67]
Sherratt, M	4	0	0	2	1[77]	1[77]
Small, P	15	0	0	25	0	0
Smith, LN	40	1[63]	1[63]	26	0	0
Stacy, R	21	0	0	5	0	0
Steel, S	18	1[72]	1[72]	5	0	0
Toeg, D	6	1[61]	1[61]	1	1[61]	1[61]
Varnam, MA	13	1[43]	1[43]	7	1[43]	1[43]
White, M	579	0	0	0	0	0

**Table 4:** summary of interventions delivered and evaluations performed (studies included in the review)

**Authors (date)**	**Intervention delivered**				**Evaluation performed**			
	
	**Who gave advice?**	**Where was advice given?**	**Referral system**	**Eligibility criteria (size of eligible population)**	**Financial**	**Non-financial, before-and-after design**	**Non-financial comp./control group**	**Qualitative**
Abbott & Hobby (1999)[79]	CAB worker	primary care or client's home	PHCT, self	all registered at 7 practices	No	Yes	Yes	Yes
Abbott & Hobby (2002)[34]	CAB worker and city council welfare rights officer	primary care	variable	(94+ practices)	No	Yes	Yes	Yes
Actions (2004)[81]	welfare rights advisers	primary care, clients' homes, telephone	self, medical staff, friends and family, voluntary and community _rganizations, social services, various other services	not reported	Yes	No	No	Yes
Bennett (1997)[82]	CAB worker	CAB office	PHCT	all registered at 3 practices	Yes	No	No	No
Borland (2004)[83, 84]	CAB worker	primary care, community hospitals, CAB offices, client's home	PHCT, self, any other agency	(Wales wide)	No	No	No	Yes
Bowran (1997)[85]	CAB worker	primary care	not reported	(n = 12500)	No	No	No	Yes
Broseley Health and Advice Partnership (2004)[86]	CAB worker	Primar care	self and all those registered at practice aged over 75 invited to take part	those registered at health centre	Yes	No	No	Yes
Bundy (2002)[87, 88]	city council welfare rights officer and CAB worker	primary care	PHCT, self	(9 practices)	Yes	No	No	No
Bundy (2003)[88]	city council welfare rights officer and CAB worker	primary care	PHCT, self, any other agency	all registered at practices covering 1/3 of those registered in Salford	Yes	No	No	No
Coppell et al (1999)[43]	welfare rights officer	primary care	PHCT, self	anyone (n = 4057)	Yes	No	No	Yes
Cornwallis & O'Neil (1998)[65]	Money advice worker	primary care	PHCT, self	all registered at practice(s) (n = 7600)	No	No	No	Yes
Derbyshire CC WRS (1997)[89]	welfare rights officer	primary care	PHCT, self	all registered at practice(s) (n = 23 039)	Yes	No	No	No
Derbyshire CC WRS (1998a)[22]	welfare rights officer	primary care	not reported	all registered at 2 practices	Yes	No	No	No
Derbyshire CC WRS (1998b)[22]	Welfare rights service worker	primary care	PHCT and targeted mailshots	(4 practices)	Yes	No	No	No
Dow & Boaz (1994)[23]	Linkage worker trained to assist in application for benefit	Clients' home or treatment facility	All individuals registered at 2 community mental health centres over 18 not currently claiming benefits, random sample of those meeting criteria at third centre, possibly eligible for benefits at screening	Screening form used – US citizen or resident alien, income <$600/month ($900 if married), one of: HIV+, 65+, blind, deaf, disabled	No	No	Yes	No
Emanuel & Begum (2000)[90]	CAB worker	primary care	PHCT, self	anyone (n = 12 601)	No	Yes	Yes	Yes
Farmer & Kennedy (2001)[91]	CAB worker	primary care, hospital	at hospitals – from ward staff to social work staff to CAB worker	not reported	No	No	No	Yes
Fleming & Golding (1997)[92]	CAB worker	primary care	not reported	all registered at 21 practices	No	No	No	Yes
Frost-Gaskin et al (2003)[66]	Mind benefit advisor	Mental health resource and day centres (primary care)	None – advisors approached as many regular attendees as possible	all regular attendees (population of those eleigible to attend = 313 510)	Yes	No	No	No
Ferguson & Simmons[93]	Community Links workers (local advice provider)	primary care	Mailshot to registered patients, GP referral	(50% of surgeries in London Borough of Newham)	No	No	Mp	Yes
Galvin et al (2000)[67, 94]	CAB worker	primary care	PHCT	(7 practices)	No	No	No	Yes
Greasley (2003)[95] and Greasley & Small (2005)[96]	12 advisors from 6 agencies	primary care	PHCT, self, any other agency	(n = 106 707)	Yes	Yes	No	Yes
Griffiths (1992)[97]	city council welfare rights officer	primary care	PHCT, self, any other agency	(2 health centres)	Yes	No	No	No
Hastie (2003)[98]	CAB worker	primary care, 2 other local locations	GP, self	not reported	Yes	No	No	Yes
High Peak CAB (1995)[99]	CAB worker	primary care	not reported	all those in town (n = 2500)	No	No	No	No
High Peak CAB (2001)[100]	CAB workers	not reported	not reported	not reported	Yes	No	No	No
High Peak CAB (2003)[101]	CAB workers	primary care	PHCT, self, other agencies	all registered at practices involved	Yes	No	No	No
Hoskins & Smith (2002)[63]	welfare rights officer	client's home	community nurses screened for attendance allowance eligibility opportunistically from their client list and referred screen positive	those >64 who in community nurses opinion were physically/mentally frail (population>64 = 1690)	Yes	No	No	Yes
Hoskins et al (in press)[102]	money advice workers	clients' homes	community nurses screened for attendance allowance eligibility from their client list and referred screen positive	those over 64 who appeared to have unmet clinical needs	Yes	No	No	No
Knight (2002)[103]	welfare benefits advisor	primary care and client's home	all aged 75+ identified through GP and sent invitation to take part	all aged 75+ in central Liverpool PCT area (n = 31 000)	No	No	No	Yes
Lancashire CC WRS (2001)[104]	welfare rights officer	client's home	all patients aged 80+ invited to take part	all registered at 3 practices 80+	No	No	No	No
Langley et al (2004)[25]	Welfare benefits advice worker	primary care, hospital, client's home, local CAB	after consent obtained, sent health assessment questionnaire. Those with score >1/5 contacted by advisor and offered advice session	over 16 with rheumatoid arthritis or osteoarthritis of knee or hip for >1 yr plus NSAID recruited from 20 practices. If >100 eligible from any practice, random sample of 100	No	No	No	No
Lishman-Peat & Brown (2002)[105]	not reported	primary care and client's home	PHCT, self	(5 practices)	Yes	No	No	Yes
MacMillan & CAB Partnership (2004)[106]	CAB workers	clients' homes, "acute and primary care locations" and cancer information centres	from nursing staff at 3 hospitals and community MacMillan nurses	cancer patients and their families	Yes	No	No	Yes
Memel & Gubbay (1999)[57]	welfare rights advisor	primary care	not reported	not reported	No	No	No	No
Memel et al (2002)[24]	CAB worker	primary care or hospital	those with RA or OA from follow up patients at rheumatology outpatients at a teaching hospital and those from two GP surgeries who had take part in other research project	diagnosis of OA or RA, being seen at outpatients or registered at participating GP, health assessment questionnaire score of 2 or more, not currently claiming attendant's allowance or disability living allowance	No	No	No	No
Middlesbrough WRU (1999)[107]	city council welfare rights officer	primary care and client's home where necessary	PHCT	all registered at practice(s) (n = 90 500)	No	No	No	No
Middlesbrough WRU (2004)[108]	welfare rights officers	primary care and clients' homes	GPs, practice receptionists, district nurses, health visitors, health and social care assessors, Macmillan nurses, social workers, age concern	those registered at practice aged over 50	Yes	No	No	No
Middleton et al (1993a)[69]	housing department welfare rights advisor	primary care	not reported	(n = 15 000)	Yes	No	No	No
Middleton et al (1993b)[69]	CAB worker	primary care	not reported	(4 practices)	Yes	No	No	No
Moffatt (2004)[109]	Welfare rights worker	client's home	invitation to take part sent to random sample of those aged 65+	random sample (n = 400+) of those aged 65+ registered at 4 practices	Yes	No	No	Yes
Moffatt et al (2004)[110, 111]	CAB worker	primary care	PHCT, self	all registered at practice	No	No	No	Yes
Paris & Player (1993)[71]	CAB worker	primary care	PHCT	(n = 64 779)	Yes	No	No	No
Reading et al (2002)[72, 80]	CAB worker	primary care	letter to all eligible families	all families registered at 3 health centres with child under 1 year	Yes	No	Yes	Yes
Roberts (1999)[112]	CAB worker	primary care, client's home, letter, telephone	PHCT, self	(5 practices)	No	No	No	Yes
Sedgefield and district AIS (2004)[113]	CAB worker	primary care	PHCT	all registered at practice(s)	No	No	No	Yes
Sherratt et al (2000)[77]	CAB worker	3 models – primary care, telephone, client's home	PHCT (GP surgery, telephone) or targeted at housebound (home visits only)	all registered at 7 or 4 practices (in-surgery and telephone advice), all housebound patients registered with GP in Gateshead (home visits)	No	No	No	Yes
Southwark CC MAS (1998)[114]	welfare rights officer	primary care	not reported	(n = 76 417)	Yes	No	No	Yes
Toeg et al (2003)[61]	CAB worker	primary care, client's home or telephone	all those eligible invited by letter from GP	registered at practice, 80 years +, living in own home (n = 12 000)	Yes	No	No	No
Vaccarello (2004)[115]	HABIT officer	client's home	invitation letters from GPs to those aged 75+	all aged 75 in Liverpool (n = 31 000)	No	No	No	Yes
Veitch (1995) GP[21]	CAB worker	primary care	not reported	(21 practices)	Yes	Yes	No	No
Veitch (1995) mental health[21]	CAB worker	health and social services sites (mental health centres)	not reported	not reported	Yes	Yes	No	No
Veitch & Terry (1993)[44]	CAB worker	primary care	PHCT	(n = 64 779)	No	No	No	No
Widdowfield & Rickard (1996)[116]	CAB worker	primary care	PHCT, self	all registered at practice(s)	No	No	No	Yes
Woodcock (2004)[117]	city council welfare rights officer	primary care	PHCT	not reported	No	No	No	Yes

CAB = Citizen's Advice Bureau; PHCT = any member of primary healthcare team; GP = general practitioner; OA = osteoarthritis; RA = rheumatoid arthritis

**Table 5:** Papers, reports and book chapters retrieved but not included in the review with reasons for exclusion

**Author (date)**	**Description of content and reason for exclusion**
Abbot & Hobby (2003)[36]	Description of service users rather than evaluation of impacts of service.
Abbott (2000)[118]	Multi-disciplinary support service for patients with mixed social and health needs with small welfare rights component but no evaluation of welfare rights component in isolation.
Abbott (2002)[37]	Discussion of where welfare rights advice fits in terms of health interventions. No evaluation of any specific intervention programme.
Alcock (1994)[119]	Discussion of potential benefits of welfare advice in primary healthcare settings and recommendations for development of such services, not evaluation of single/multiple project(s)
Barnes (2000)[120]	Citizens advice service for patients at a long stay psychiatric hospital – including a limited amount of welfare rights advice. No specific evaluation of welfare rights advice component.
Barnsley Community Legal Service Partnership (2003)[121]	Very brief mention of a welfare rights advice project in primary care within a larger report – no evaluation of service.
Bebbington & Unell (2003)[122]	Description of a multidisciplinary telephone advice line for older people with some evaluation of use. No evaluation of welfare rights advice component.
Bebbington et al (?year)[123]	Description of a multidisciplinary telephone advice line for older people with some evaluation of use. No evaluation of welfare rights advice component.
Bird (1998)[124]	Audit of CAB services for those with mental illness – not evaluation of any specific intervention programme delivered in a healthcare setting.
Buckle (1986)[125]	Discussion of eligibility for various benefits. No evaluation of specific intervention.
Bundy (2001)[39]	Brief description of 'The Health and Advice Project' – full evaluation report included in review
Burton & Diaz de Leon (2002)[126]	Review of a number of welfare advice services but only service for which any outcomes are report does not appear to have been delivered in a healthcare setting.
Clarke et al (2001)[127]	Multidisciplinary service to provide advice and support to individuals and families with complex social and health problems – including welfare rights advice. No specific evaluation of welfare rights advice component.
Craig et al (2003)[128]	Review and primary research on the impact of addition welfare benefit income in older people – not specifically of welfare rights advice delivered in a healthcare setting.
Dowling et al (2003)[129]	Systematic review of effectiveness of financial benefits in reducing inequalities in child health with limitation to randomised controlled trials. Not evaluation of welfare rights advice.
Emanuel (2002)[130]	Description of service rather than evaluation of impacts of service.
Ennals (1990)[131]	Discussion of importance of welfare benefits in relation to health and eligibility for benefits.
Ennals (1993)[74]	Editorial relating to article (Paris and Player, 1993) included in review
Evans (1998)[132]	Report of client profile, sources of referrals and problems raised at a welfare rights advice service in primary care. No evaluation of effect on clients.
Forrest (2003)[133]	Very brief mention of a welfare rights advice project in primary care within a larger report – no evaluation of service.
Gask et al (2000)[134]	Very brief mention of a welfare rights advice project in primary care within a larger report – no evaluation of service.
Greasley & Small (2002)[135]	A review of previously published work on welfare rights advice delivered in primary care. Not an evaluation of a specific intervention.
Greasley (2005)[136]	Discussion of the process of videoing interviews that happened to be with users of a welfare rights advice service in primary healthcare. No evaluation of the impact of the intervention service itself.
Green (1998)[137]	Description of eligibility for benefits whilst an in-patient.
Green et al (2004)[138]	Review of health impact assessments in a variety of areas with very limited mention of Longworth et al (2003)
Harding et al (2002)[38]	Audit of provision of welfare rights advisors in general practices and perceived impact of these facilities on the primary healthcare team. No evaluation of any specific programme on clients.
Hobby & Abbott (1999)[78]	Brief description of 'The Health and Advice Project' – full evaluation report included in review
Hobby et al (1998)[15]	A survey of CAB offering outreach in primary care settings with collation of some information. Limited data on impacts of advice not included in other, primary, reports.
Hoskins et al (2000)[64]	Discussion of potential importance of welfare benefits advice for health with proposal that nurses could become involved in giving advice. No actual intervention described or evaluated.
Jarman (1985)[45]	Description of computer programme to help determine eligibility for various welfare benefits. No evaluation of impact of programme.
Kalra et al (2003)[48]	Methods of family planning _ounseling, not welfare rights advice related.
Longworth et al (2003)[139]	Discussion of potential, rather than actual, impact of service
NACAB (1999)[10]	Magazine type articles on various different studies with case studies, not evaluation of single/multiple project(s)
Norowska (2004)[62]	Description of delayed application for and provision of attendance allowance. No intervention to improve take-up discussed.
Okpaku (1985)[140]	Audit of mentally ill people applying for benefit and problems they encounter. No intervention programme to provide advice with claiming.
Pacitti & Dimmick (1996)[56]	Descriptive study of extend and correlates of underclaiming of welfare benefits amongst individuals with mental illness.
Powell et al (2004)[68]	Financial evaluation of welfare rights advice programme with repetition of financial impacts for clients of data in Langley et al (2004) and Memel et al (2004)
Reid et al (1998)[141]	Assessment of staff awareness and involvement in an ongoing welfare rights advice project in primary care. No evaluation of impact of service on users.
Riverside Advice Ltd (2004)[142]	Report of welfare rights project for those with mental illnesses. No evaluation of impact of service on users.
Scully (1999)[143]	Report of training programme for welfare rights advisors working within primary care settings, not evaluation of a specific service.
Searle (2001)[144]	Description of a multidisciplinary telephone advice line for older people. No evaluation of welfare rights advice component.
Sherr et al (2002)[145]	Audit of current practice in three London boroughs with exploration of attitudes to potential services, not evaluation of service in place.
Stenger (2003)[35]	Discussion of moving from welfare to work, not of advice to help claim welfare benefits.
Strachan (1995)[146]	Proceedings of a conference with descriptions but no evaluations of welfare rights advice services in healthcare settings.
Tameside MBC [33, 147]	Description of rationale for service and recommendations for the future, not evaluation of service
Thomson et al (2004)[95]	Discussion of problems involved in rigorous scientific evaluation of social interventions – including welfare rights advice – but no evaluation of specific intervention.
Venables (2004)[148]	Annual report of welfare rights service not based in a healthcare setting.
Watson (2000)[149]	Multidisciplinary intervention project with small welfare rights component but no evaluation of welfare rights component in isolation.
Waterhouse (1996)[150]	Profile of users of a welfare rights advice service in primary care, along with advice sought, service provided and discussion of logistic issues. No evaluation of effect on clients.
Waterhouse (2003)[151]	Report on logistical problems and solutions to setting up welfare advice service in primary care. No evaluation of effect on clients.
Waterhouse and Benson (2002)[152]	Background paper proposing establishment of a welfare rights service within a PCT. No evaluation of new project.
West Berkshire CAB (2004)[153]	Report of service activity and financial statement – no evaluation of service.
Williams (1982)[154]	Description of a hospital based services. Evaluation limited to type of contacts and activity engaged in by welfare advisor.

**Table 6:** health and social outcomes (validated measurement instruments), studies with a control or comparison group (studies included in the review)

**Authors (date)**	**Outcome measure**	**Nature of control/comparison group**	**Random allocation?**	**Control group N at baseline**	**Intervention group N at baseline**	**Control group mean score at baseline**	**Intervention group mean score at baseline**	**Follow up period**	**Control N at follow up**	**Intervention N at follow up**	**Control group mean score at follow up**	**Intervention group man score at follow up**	p-value*
Abbott & Hobby (1999)[79]	SF36 physical functioning (change in score)	Those whose income didn't increase following advice allocated to comparison group	No	20	48	NR	NR	6 months	20	48	0	2.4	p > 0.05
	SF36 role functioning physical (change in score)		No	20	48	NR	NR	6 months	20	48	-2.5	2.1	p > 0.05
	SF36 bodily pain (change in score)		No	20	48	NR	NR	6 months	20	48	1	-0.5	p > 0.05
	SF36 general health (change in score)		No	20	48	NR	NR	6 months	20	48	2.5	3.3	p > 0.05
	SF36 vitality (change in score)		No	20	48	NR	NR	6 months	20	48	-7	7.7	**p = 0.001**
	SF36 social functioning (change in score)		No	20	48	NR	NR	6 months	20	48	-1.3	2.9	p > 0.05
	SF36 role functioning emotional (change in score)		No	20	48	NR	NR	6 months	20	48	8.3	14.6	p > 0.05
	SF36 mental health (change in score)		No	20	48	NR	NR	6 months	20	48	-4.8	7.2	**p = 0.019**

Abbott & Hobby (2002)[34]	SF36 physical functioning	Those whose income didn't increase following advice allocated to comparison group	No	50	150	34	29.5	6 months	50	150	34.2	30.6	p = 0.65
	SF36 physical functioning		No	50	150	34	29.5	12 months	50	150	37.7	28.9	p = 0.17
	SF36 role functioning physical		No	50	150	15.5	18.9	6 months	50	150	24.5	28.1	p = 0.5
	SF36 role functioning physical		No	50	150	15.5	18.9	12 months	50	150	27	26	p = 0.74
	SF36 bodily pain		No	50	150	29.2	34.8	6 months	50	150	30	43.1	**p = 0.013**
	SF36 bodily pain		No	50	150	29.2	34.8	12 months	50	150	36.4	39.4	p = 0.71
	SF36 general health		No	50	150	35.6	31.7	6 months	50	150	34	32.3	p = 0.59
	SF36 general health		No	50	150	35.6	31.7	12 months	50	150	32.3	32.1	p = 0.35
	SF36 vitality		No	50	150	33.2	28.7	6 months	50	150	28.4	32.3	p = 0.13
	SF36 vitality		No	50	150	33.2	28.7	12 months	50	150	29.2	28.4	p = 0.26
	SF36 social functioning		No	50	150	45.8	42.3	6 months	50	150	52.5	50.2	p = 0.58
	SF36 social functioning		No	50	150	45.8	42.3	12 months	50	150	54.6	49.2	p = 0.58
	SF36 role functioning emotional		No	50	150	48.7	40.8	6 months	50	150	36.7	51.7	p = 0.17
	SF36 role functioning emotional		No	50	150	48.7	40.8	12 months	50	150	42.7	52.2	**p = 0.02**
	SF36 mental health		No	50	150	57.1	53	6 months	50	150	56	55.9	p = 0.84
	SF36 mental health		No	50	150	57.1	53	12 months	50	150	56	58.3	**p = 0.03**

Emanuel & Begum (2000)[90]	HADS anxiety	Those whose income didn't increase following advice allocated to comparison group	No	28	12	12.03	12	9 months	28	13	11.14	12.58	p > 0.05
	HADS depression		No	28	12	8.21	9.75	9 months	28	13	7.86	9.33	p > 0.05
	MYMOP symptom 1		No	28	12	4.48	4.64	9 months	28	13	3.86	4.36	p > 0.05
	MYMOP symptom 2		No	28	12	3.59	4.67	9 months	28	13	2.41	5.33	p > 0.05
	MYMOP activity		No	28	12	4.17	5.7	9 months	28	13	3.83	5	p > 0.05
	MYMOP wellbeing		No	28	12	3.86	4.55	9 months	28	13	3.14	4.65	p > 0.05
	MYMOP profile		No	28	12	4.53	4.28	9 months	28	13	3.44	4.79	p > 0.05

	GP consultations in last 9 months	Control identified as next in individual on practice register matched for age and sex.	No	39	39	70	187	9 months	39	39	111	165	p > 0.05
	prescriptions in last 9 months		No	39	39	122	239	9 months	39	39	146	278	p > 0.05
	referrals to secondary care in last 9 months		No	39	39	3	21	9 months	39	39	5	18	p > 0.05
	Visits to A&E in last 9 months		No	39	39	0	1	9 months	39	39	2	0	p > 0.05
	practice nurse contacts in last 9 months		No	39	39	13	12	9 months	39	39	6	11	p > 0.05
	home visits in last 9 months		No	39	39	5	3	9 months	39	39	1	3	p > 0.05
	out of hours calls in last 9 months		No	39	39	2	3	9 months	39	39	3	5	p > 0.05
	social service referrals in last 9 months		No	39	39	0	0	9 months	39	39	0	0	p > 0.05
	cervical cancer screening in last 9 months		No	39	39	1	1	9 months	39	39	5	7	p > 0.05

Reading et al (2002)[72]	Edinburgh postnatal depression scale	Six practices recruited – three allocated to intervention group, three to control group.	Yes	173	88	7.7	9.7	NR	153	66	7.1	8.1	p > 0.05
	Prevalence of maternal smoking		Yes	173	88	25	34	NR	153	66	20	36	p > 0.05
	Maternal non-routine GP visits per year		Yes	173	88	NR	NR	NR	153	66	3.1	3.5	p > 0.05
	Maternal prescriptions		Yes	173	88	NR	NR	NR	153	66	2.4	2.1	p > 0.05
	Child general health "very good"		Yes	173	88	NR	NR	NR	153	66	51	44	p > 0.05
	Child more than 2 minor illnesses in last 3 months		Yes	173	88	NR	NR	NR	153	66	18	22	p > 0.05
	Child accident requiring attention in last year		Yes	173	88	NR	NR	NR	153	66	10	6	p > 0.05
	Child behaviour problems		Yes	173	88	NR	NR	NR	153	66	5	10	p > 0.05
	Child sleeping problems		Yes	173	88	12	13	NR	153	66	12	14	p > 0.05
	Child currently breast fed or stopped aged >4 months		Yes	173	88	31	31	NR	153	66	23	17	p > 0.05
	Child non-routine GP visits per year		Yes	173	88	NR	NR	NR	153	66	4.2	4.2	p > 0.05
	Child prescriptions		Yes	173	88	NR	NR	NR	153	66	2.4	2	p > 0.05

Veitch (1995) GP[21]	NHP total score	Those identified by control practices who would have been referred had service been available.	No	5	5	NR	NR	NR	5	5	NR	NR	p > 0.05
	NHP energy		No	5	5	NR	NR	NR	5	5	NR	NR	p > 0.05
	NHP pain		No	5	5	NR	NR	NR	5	5	NR	NR	p > 0.05
	NHP emotional reaction		No	5	5	NR	NR	NR	5	5	NR	NR	p > 0.05
	NHP sleep		No	5	5	NR	NR	NR	5	5	NR	NR	p > 0.05
	NHP social isolation		No	5	5	NR	NR	NR	5	5	NR	NR	p > 0.05
	NHP physical mobility		No	5	5	NR	NR	NR	5	5	NR	NR	p = 0.09

Veitch (1995) mental health[21]	NHP total score	Those identified by control mental health centres who would have been referred had service been available.	No	12	36	NR	NR	NR	12	18	NR	NR	p = 0.4588
	NHP energy		No	12	36	NR	NR	NR	12	18	NR	NR	p = 0.2312
	NHP pain		No	12	36	NR	NR	NR	12	18	NR	NR	**p = 0.0700**
	NHP emotional reaction		No	12	36	NR	NR	NR	12	18	NR	NR	**p = 0.0466**
	NHP sleep		No	12	36	NR	NR	NR	12	18	NR	NR	p = 0.3095
	NHP social isolation		No	12	36	NR	NR	NR	12	18	NR	NR	p = 0.4872
	NHP physical mobility		No	12	36	NR	NR	NR	12	18	NR	NR	p = 0.1312

Dow & Boaz (1994)[23]	applied for award	Random allocation to intervention/control group	Yes	389	387	0	0	6 months	311	303	20	63	**p < 0.001**
	applied for award		Yes	389	387	0	0	8 months	311	303	26	67	**p < 0.05**
	applied for award		Yes	389	387	0	0	11 months	311	303	26	67	**p < 0.05**
	received award		Yes	389	387	0	0	6 months	311	303	8	17	**p < 0.05**
	received award		Yes	389	387	0	0	8 months	311	303	12	22	**p < 0.05**
	received award		Yes	389	387	0	0	11 months	311	303	13	23	**p < 0.051**

*comparison of change in score in intervention group with change in score in control or comparison group; SF36 = short form 36; MYMOP = Measure Yourself Medical Outcome Profile scale; GP = general practitioner; A&E = accident and emergency; NHP = Nottingham Health Profile; NR = not reported

**Table 7:** Quantitative scalar health outcomes, before and after studies (studies included in the review)

**Authors (date)**	**Outcome measure**	**Baseline N**	**Baseline mean score**	**Follow up period**	**Follow up N**	**Follow up mean score**	p-value*
Abbott & Hobby (1999)[79]	SF36 physical functioning	48	20.8	before vs after income increase	48	23.1	p > 0.05
	SF36 role functioning physical	48	12.5	before vs after income increase	48	14.6	p > 0.05
	SF36 bodily pain	48	25.5	before vs after income increase	48	24.9	p > 0.05
	SF36 general health	48	26.7	before vs after income increase	48	30	p > 0.05
	SF36 vitality	48	20.8	before vs after income increase	48	28.5	**p = 0.002**
	SF36 social functioning	48	29.4	before vs after income increase	48	32	p > 0.05
	SF 36 role functioning emotional	48	36.8	before vs after income increase	48	51.4	**p = 0.037**
	SF36 mental health	48	45.9	before vs after income increase	48	53.1	**p = 0.005**

Abbott & Hobby (2002)[34]	SF36 physical functioning	345	35.8	6 months	244	31.5	p > 0.05
	SF36 physical functioning	345	35.8	12 months	200	30.6	p > 0.05
	SF36 role functioning physical	345	22.8	6 months	244	18.9	p > 0.05
	SF36 role functioning physical	345	22.8	12 months	200	18	p > 0.05
	SF36 bodily pain	345	35.7	6 months	244	33.2	p > 0.05
	SF36 bodily pain	345	35.7	12 months	200	33.4	p > 0.05
	SF36 general health	345	34.8	6 months	244	32.9	p > 0.05
	SF36 general health	345	34.8	12 months	200	32.6	p > 0.05
	SF36 vitality	345	31.3	6 months	244	29.9	p > 0.05
	SF36 vitality	345	31.3	12 months	200	29.8	p > 0.05
	SF36 social functioning	345	40.9	6 months	244	42.5	p > 0.05
	SF36 social functioning	345	40.9	12 months	200	43.2	p > 0.05
	SF36 role functioning emotional	345	40.9	6 months	244	40.4	p > 0.05
	SF36 role functioning emotional	345	40.9	12 months	200	42.8	p > 0.05
	SF36 mental health	345	51.7	6 months	244	53.1	p > 0.05
	SF36 mental health	345	51.7	12 months	200	54	p > 0.05

Emanuel & Begum (2000)[90]	HADS anxiety	40	12.03	9 months	40	11.58	p > 0.05
	HADS depression	40	8.68	9 months	40	8.3	p > 0.05
	MYMOP symptom 1	31	4.58	9 months	31	4.1	p > 0.05
	MYMOP symptom 2	25	3.92	9 months	25	3.48	p > 0.05
	MYMOP activity 1	27	4.67	9 months	27	4.26	p > 0.05
	MYMOP wellbeing	31	4.13	9 months	31	3.71	p > 0.05
	MYMOP profile	31	4.45	9 months	31	3.94	p > 0.05

Greasley (2003)[95]	SF36 physical functioning	22	39.09	6 months	22	48.64	p > 0.05
	SF36 physical functioning	22	39.09	12 months	22	57.50	p > 0.05
	SF36 role functioning physical	22	30.11	6 months	22	36.36	p > 0.05
	SF36 role functioning physical	22	30.11	12 months	22	40.34	p > 0.05
	SF36 bodily pain	22	30.45	6 months	22	25.91	p > 0.05
	SF36 bodily pain	22	30.45	12 months	22	29.18	p > 0.05
	SF36 general health	22	22.90	6 months	22	31.09	**p < 0.002**
	SF36 general health	22	22.90	12 months	22	33.59	**p < 0.076**
	SF36 vitality	22	25.28	6 months	22	26.98	**ANOVA across 3 time points, p < 0.079**
	SF36 vitality	22	25.28	12 months	22	33.52	
	SF36 social functioning	22	34.09	6 months	22	43.75	**ANOVA across 3 time points, p < 0.077**
	SF36 social functioning	22	34.09	12 months	22	43.75	
	SF36 role functioning emotional	22	34.85	6 months	22	47.72	p > 0.05
	SF36 role functioning emotional	22	34.85	12 months	22	39.77	p > 0.05
	SF36 mental health	22	37.14	6 months	22	42.85	p > 0.05
	SF36 mental health	22	37.14	12 months	22	47.86	**p < 0.076**

Greasley (2003)[95] cont.	HADS anxiety	22	13.31	6 months	22	11.73	**ANOVA across 3 time points, p < 0.051**
	HADS anxiety	22	13.31	12 months	22	11.36	
	HADS depression	22	10.59	6 months	22	10.41	p > 0.05
	HADS depression	22	10.59	12 months	22	9.59	p > 0.05

Veitch (1995) – GP[21]	NHP total score	52	Not reported	6 months	52	Not reported	p-0.6344
	NHP energy	52	Not reported	6 months	52	Not reported	p = 0.3970
	NHP pain	52	Not reported	6 months	52	Not reported	p = 0.8368
	NHP emotional reactions	52	Not reported	6 months	52	Not reported	p = 0.4249
	NHP sleep	52	Not reported	6 months	52	Not reported	p = 0.3138
	NHP social isolation	52	Not reported	6 months	52	Not reported	p = 0.9011
	NHP physical mobility	52	Not reported	6 months	52	Not reported	p = 0.8489

Veitch (1995) – mental health[21]	NHP total score	52	Not reported	6 months	52	Not reported	p = 0.1084
	NHP energy	52	Not reported	6 months	52	Not reported	p = 0.3359
	NHP pain	52	Not reported	6 months	52	Not reported	**p = 0.0127**
	NHP emotional reactions	52	Not reported	6 months	52	Not reported	**p = 0.0333**
	NHP sleep	52	Not reported	6 months	52	Not reported	p = 0.1309
	NHP social isolation	52	Not reported	6 months	52	Not reported	p = 0.8928
	NHP physical mobility	52	Not reported	6 months	52	Not reported	p = 0.2061

*comparison of follow up versus baseline score; SF36 = short form 36; MYMOP = Measure Yourself Medical Outcome Profile scale; HADS = Hospital Anxiety and Depression Scale; NHP = Nottingham Health Profile

**Table 8:** Quantitative non-scalar health and social outcomes, studies without a control or comparison group (studies included in the review)

**Authors (date)**	**Sample size and composition**	**Sample selection strategy**	**Data collection method**	**Summary of results**
Abbott & Hobby (1999)[79]	48 clients	all clients whose income increased as a result of the advice	structured interview	69% felt increase in income "affected how they felt about life and/or that their health had improved"
Borland (2004)[83, 84]	1088 clients	all clients asked to complete questionnaire	postal questionnaire	88% felt better after seeing the advice worker
Broseley Health and Advice Partnership (2004)[86]	unspecified number of clients	not reported	postal questionnaire	100% "felt less worried or stressed" following the advice 75% "had more money to buy food or provide heating" following the advice 75% "felt better in themselves" following the advice
Hastie (2003)[98]	86 clients	not reported	postal questionnaire	87% thought the service "made a positive difference to them" 83% "felt less worried, calmer and supported" following the advice 60% "felt their health had improved" following the advice 53% "felt that their housing situation had improved" following the advice
Lishman-Peat & Brown (2002)[105]	34 clients	not reported	structured interview	73% "felt happier having been helped by ad advisor, even if that help did not result in extra income"
Sedgefield and district AIS (2004)[113]	33 clients	not reported	postal questionnaire	73% felt advice had "improved quality of life"
Vaccarello (2004)[115]	unspecified number of clients	10% random sample of clients invited to take part	postal questionnaire	98% felt service "had improved their quality of life" 91% said the service "had helped them to keep independent and remain in their own home" 83% "felt they were able to manage more safely in their homes" following the advice 77% felt they "cope better with their day-to-day living" following the advice
Ferguson & Simmons[93]	unspecified number of clients	not reported	not reported	46% felt "less anxious or worried" after seeing the advisor 11% "reported an improvement in their health" 13% "reported that they could now afford a better diet" 13% "stated that they could afford increased heating" as a result of the advice

**Table 9:** Quality of qualitative studies (studies included in the review)

**Authors (date)**	**Sample Size**	**Sample composition**	**Sample selection strategy**	**Data collection method**	**Analytical method**
Abbott & Hobby (2002)[34]	6	clients	illustrative of "complex interactions between social situation, income and health"	interviews	development of case studies
Actions (2004)[81]	Not stated	clients	Not stated	questionnaire with free text	non stated – verbatim reporting of free text comments
Bowran (1997)[85]	25	17 successful claimants, 7 unsuccessful claimants	all those seen in 1996 invited to take part, 43 consented, purposefully sampled	unstructured interviews	grounded theory
Emanuel & Begum (2000)[90]	10	10 clients	5 users whose HADS/MYMOP improved, 5 users whose HADS/MYMOP didn't improve/worsened	semi-structured interviews	thematic analysis
Farmer & Kennedy (2001)[91]	8	4 clients after advice given, 4 clients before and after advice given	clients seen after chosen by random selection, clients seen before and after approached in waiting room and asked to take part	semi-structured interviews	development of case studies and inductive thematic analysis
Fleming & Golding (1997)[92]	27	clients	all clients who gave consent	semi-structured interviews	not stated – description of apparently important areas reported
Galvin et al (2000)[67, 94]	10	clients	service users those with multiple and complex needs	"focused interviews"	illuminative evaluation, thematic content analysis
Knight (2002)[103]	28	service users	not stated	focus groups and telephone unstructured interviews	thematic analysis
MacMillan & CAB Partnership (2004)[106]	38	clients	Those clients who gave permission to be contacted for research	telephone interview	not stated – verbatim reporting of comments given
Moffatt et al (2004)[70]	11	all white, 7 women, age range 46–76 years, all unemployed/retired/unable to work, all chronic health problems, 8 never used welfare advice before	purposeful of those who benefited financially	semi-structured interviews	establish analytical categories, grouping into overarching key themes
Moffatt (2004)[109]	25	14 in intervention arm, 14 female, mean age 75	purposeful to get those who did and didn't receive intervention and those who did and didn't benefit financially	semi-structured interviews	development of conceptual framework and thematic charting
Reading et al (2002)[72]	10	5 service users and 5 non-service users who were eligible and expressed debt concerns at start of project	random selection of two groups represented	semi-structure interviews	modified grounded theory with more descriptive approach
Sherratt et al (2000)[77]	41	13 patients	4 patients randomly chosen per month and invited to take part	semi-structured interviews with clients, focus groups with staff	thematic analysis
Woodcock (2004)[117]	Not stated	clients	all clients seen sent satisfaction questionnaire	postal questionnaire with free text	not stated – verbatim reporting of few text comments

**Table 10:** Quantitative financial outcomes (studies included in review where data provided)

**Authors (date)**	**Number of clients seen**	**Total lump sum/one off payments gained**	**Mean lump sum/one off payments per client seen**	**Recurring benefits gained (per year)**	**Mean recurring benefits (per year) per client seen**
Bennett (1997)[82]	49	£28 121.00	£573.898	£41 860.00	£854.29
Bundy (2002)[87]	561	£183 147.00	£326.47	£762 042.00	£1358.36
Bundy (2003)[88]	818	£261 231.00	£319.35	£474 587.00	£580.18
Coppell et al (1999)[43]	270	£15 863.00	£58.75	£28 028.00	£103.81
Cornwallis & O;Neill (1997)[65]	102	£66 785.00	£654.75	not reported	not reported
Derbyshire CC WRS (1997)[89]	428	£73 643.07	£172.06	£527 352.90	£1232.13
Derbyshire CC WRS (1998a)[22]	480	£117 405.20	£244.59	£573 995.20	£1195.82
Derbyshire CC WRS (1998b)[22]	290	£56 967.87	£196.44	£374 630.40	£1291.83
Frost-Gaskin et al (2003)[66]	153	£60 323.34	£394.27	£281 805.80	£1841.87
Greasley (2003)[95] & Greasley and Small (2005)[96]	2484	£431 198.00	£173.59	£1 940 543.00	£781.22
Griffiths (1992)[97]	157	£32 708.00	£208.33	£87 131.20	£554.98
Hastie (2003)[98]	492	£39 688.00	£80.67	£173 108.00	£351.85
High Peak CAB (1995)[99]	39	not reported	not reported	£38 646.40	£990.93
High Peak CAB (2001)[100]	236	£9 069.74	£38.43	£24 934.52	£105.65
High Peak CAB (2003)[101]	156	£4765.63	£30.55	£60 201.96	£385.91
Hoskins et al (in press)[102]	630	£119 515.44	£189.71	£1 016 908.70	£1 614.14
Memel & Gubbay (1999)[57]	46	not reported	not reported	£73 872.00	£1605.91
Memel et al (2002)[24]	19	not reported	not reported	£38 725.00	£2038.16
Middlesbrough WR (1999)[107]	272	not reported	not reported	£473 053.00	£1739.17
Middleton et al (1993a)[69]	52	£10 393.00	£199.87	£14 359.00	£276.13
Middleton et al (1993b)[69]	583	£12 559.80	£21.54	£8 373.20	£14.36
Moffatt (2004)[109]	25	£5 766.00	£230.64	£37 442.08	£1497.68
Paris & Player (1993)[71]	150	£3 371.00	£22.47	£54 929.58	£366.20
Reading et al (2002)[72]	23	£4 389.00	£190.83	£6 480.00	£281.74
Southwark CC MAC (1998)[114]	621	£160 593.00	£258.60	£390 500.00	£628.82
Vaccarello (2004)[115]	206	£11 433.00	£55.50	£137 819.00	£669.02
Veitch (1995)[21] – mental health	35	£16 122.90	£460.65	£25 581.40	£730.90
Veitch (1995)[21] – GP	37	£28 783.69	£777.94	£74 025.64	£2000.69
Widdowfield & Rickard (1996)[116]	106	not reported	not reported	£183 790.20	£1733.87
**Totals**		**£1 753 843 and 9038 clients, mean = £194 per client**		**£7 864 910 and 9418 clients, mean = £832 per year per client**	

CAB = Citizen's Advice Bureau
